# A Case of Recurrent Mesocolon Myxoid Liposarcoma and Review of the Literature

**DOI:** 10.1155/2013/692754

**Published:** 2013-11-07

**Authors:** Amar M. Eltweri, Gianpiero Gravante, Sarah Louise Read-Jones, Sonpreet Rai, David J. Bowrey, Ian Gordon Haynes

**Affiliations:** ^1^Leicester Royal Infirmary, University Hospital of Leicester, Leicester LE1 5WW, UK; ^2^Department of Colorectal Surgery, George Eliot Hospital, Nuneaton CV10 7DJ, UK; ^3^Department of Histopathology, George Eliot Hospital, Nuneaton CV10 7DJ, UK

## Abstract

*Background*. Liposarcoma is the second most common soft tissue sarcoma affecting predominantly the retroperitoneal space and extremities. Mesenteric liposarcoma is uncommon and occurs in the small bowel mesentery. In this paper we report the case of a recurrent mesocolon myxoid liposarcoma manifesting 6 years from the initial right hemicolectomy for the primary tumour. *Case Report*. A 41-year-old female presented with a 4-day history of signs and symptoms indicative of small bowel obstruction, subsequently confirmed on plain abdominal X-ray. In 2006 she underwent a right hemicolectomy for a myxoid liposarcoma of the mesentery. The patient was initially managed conservatively; however she showed no signs of improvement and was taken to theatre for an exploratory laparotomy and division of adhesional bands. During this procedure an incidental finding of a dark purple, smooth pelvic mass was identified with similar macroscopic appearance to that of splenic tissue. Histological examination revealed a recurrent mesocolon myxoid liposarcoma. *Conclusion*. Mesocolon myxoid liposarcoma is a rare soft tissue neoplastic pathology and carries a high risk of recurrence. Therefore, a symptomatic patient with a previous history of primary liposarcoma excision should be treated with a high index of suspicion and a longer period of followup should be considered.

## 1. Introduction

Liposarcoma is a group of malignancies of mesenchymal origin that arise from adipose tissue. The incidence peaks in the fourth to sixth decades of life [1]. CT and MRI are important imaging modalities in determining tissue characteristics, the size of the tumour, and invasion into surrounding structures [2]. When feasible, the main treatment is surgical resection followed by adjuvant chemotherapy and/or radiotherapy [3]. Important prognostic factors include the histological classification and tumour site and size [4] while positive surgical margins are key predictors for local recurrence [3].

Liposarcomas are usually located in the lower limbs of adults [1, 4], rarely in the small bowel mesentery and even less frequently in the mesocolon. In this report, we present the case of a recurrent mesocolon myxoid liposarcoma manifesting six years from the initial right hemicolectomy and review the literature regarding mesenteric liposarcomas.

## 2. Case Report

A 41-year-old female presented to the emergency department with a four-day history of signs and symptoms indicative of abdominal obstruction. Her past medical history included hypothyroidism due to autoimmune thyroiditis, managed with levothyroxine. In 2006 she underwent a right hemicolectomy for a myxoid liposarcoma grade 1-2 infiltrating the small and large bowels. The specimen weighed almost 3 kilograms with dimensions 25 × 22 × 11 cm. The removal was radical with free margins and an intact tumour capsule. Postoperatively, no adjuvant therapy was indicated and close followup was recommended.

**Figure 1:**
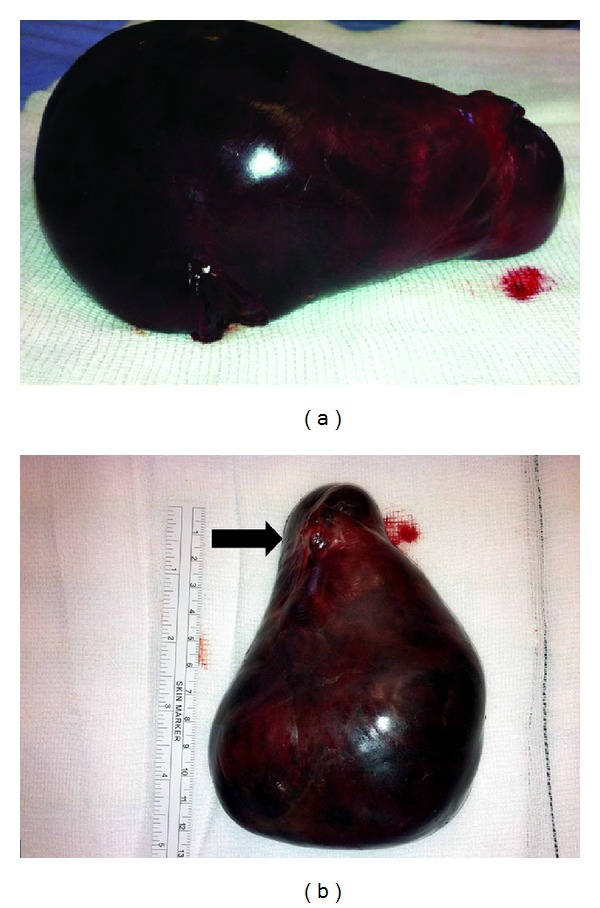
Macroscopic appearance of the myxoid liposarcoma: a dark purple smooth mass measuring 12.5 × 11 × 6 cm that weighs 326 grams similar to splenic tissue. The black arrow indicates the stalk by which the liposarcoma was attached to the pelvic wall.

During the current admission she was dehydrated, haemodynamically stable, and apyrexial. Abdominal examination revealed a distended abdomen, tympanic to percussion, with no signs of peritonism or abdominal wall hernias. Blood investigations showed a raised urea (8.7 mmol/L) and WCC 14.2 × 10^9^. Abdominal X-ray revealed grossly dilated small bowel loops. The initial treatment was conservative with nil by mouth, intravenous fluid resuscitation, nasogastric tube, and urinary catheter for fluid balance. After twenty-four hours the patient showed no signs of improvement and underwent an exploratory laparotomy. An adhesional band was found to be the cause of the small bowel obstruction and was divided. The entire small bowel was viable and no evidence of intra-abdominal or peritoneal metastasis was identified. However, a dark purple, smooth pelvic mass was found attached to the pelvic wall by a small stalk with similar macroscopic appearance to that of splenic tissue (Figure 1). The mass was carefully detached off the pelvic wall and sent for final histological analysis.

**Figure 2:**
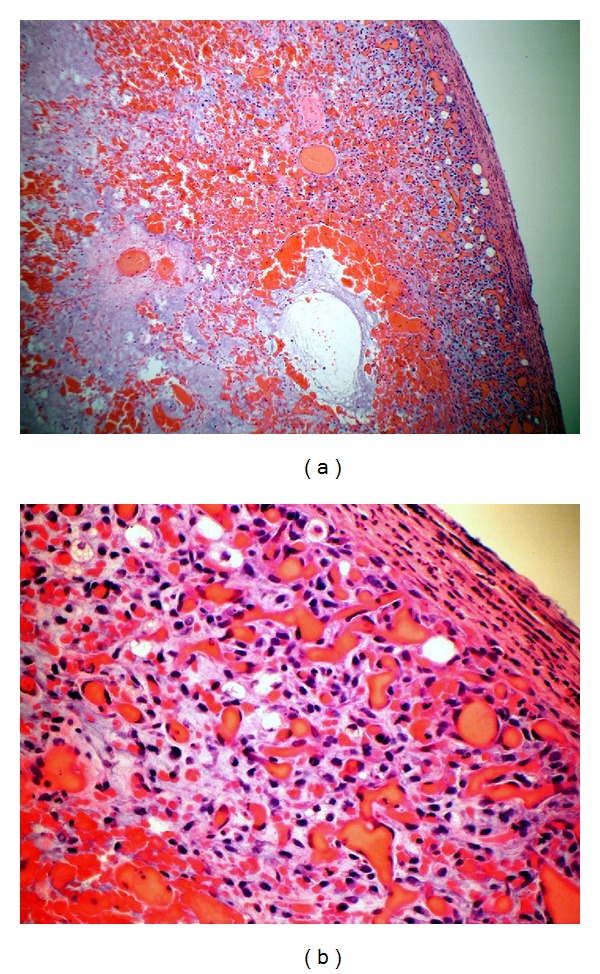
(a) Haemorrhagic infarcted tissue with background myxoid material and some viable cells identified at the periphery (haematoxylin-eosin, 10x). (b) Both lipoblasts and round cells at the periphery of the tumour (haematoxylin-eosin, 40x).

**Figure 3:**
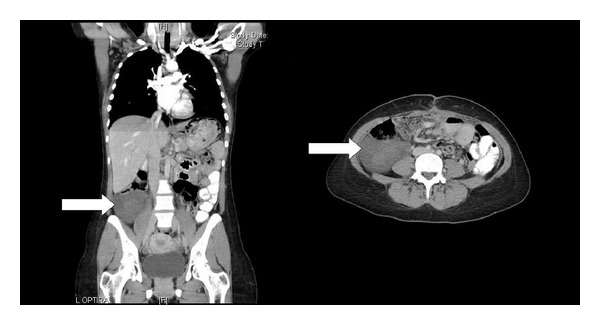
Frontal (*left panel*) and transverse (*right panel*) views of the CT scan performed postoperatively. A well-defined oval hypodense uniform mass is appreciated at the right iliac fossa measuring 10 × 8.4 cm with a central rounded higher density soft tissue area within it (white arrows).

After the laparotomy the patient had an uncomplicated recovery and was discharged home on the eighth postoperative day. Histology confirmed a recurrence of the previous myxoid liposarcoma (Figure 2). One month later a CT scan of the chest, abdomen, and pelvis showed a new well-defined oval hypodense mass in the right iliac fossa adjacent to the anastomotic surgical sutures site that was suspicious for recurrence (Figure 3). In light of these findings, the patient has been referred to the regional sarcoma centre for further management.

## 3. Discussion

According to the World Health Organisation classification of tumours [5], liposarcomas are divided into well-differentiated/dedifferentiated, pleomorphic, myxoid/round cell, and mixed type liposarcoma (Table 1). Myxoid liposarcoma is a mesenchymal malignant tumour composed of uniform round to oval primitive nonlipogenic mesenchymal cells and a number of small signet-ring lipoblasts in a myxoid stroma with a characteristic branching vascular pattern. It is also called round cell liposarcoma and it is the second most common liposarcoma subtype. It usually presents during the fourth and fifth decades of life as a large painless mass in the deep soft tissue of the extremities. More than two-thirds of the myxoid liposarcoma cases occur within the muscles of the thigh and rarely occur in the subcutaneous tissues or the retroperitoneum. The presence of necrosis usually indicates a poor prognosis [5]. Myxoid liposarcoma is likely to recur locally and one-third of cases develop distant metastasis [5]. The sites of reported metastasis and/or recurrence of liposarcoma were local, cardiac, hepatic, mesenteric, bone, and pulmonary [3, 6, 7]. The overall survival ranges between 6 and 20 years [8].

Through literature review, only five mesenteric liposarcomas of the mesocolon have been published to date [3, 9–11]. Among them only one was recurrent [12]; therefore our case represents the second recurrence of a myxoid liposarcoma arising from the mesocolon reported in the literature. Benedict first described mesenteric liposarcomas in 1946 as a recurrent liposarcoma of the transverse mesocolon. Since then, various cases have been presented (Table 2). Mesenteric liposarcomas affect both the male and female sex equally and are more evident during the fifth to seventh decade of life. It may present in any age group and has been reported in patients as young as 15 years old [13]. The clinical presentation varies and includes abdominal pain, distension, palpable mass, constipation, vomiting, and weight loss (Table 2). CT and MRI investigations add important data for the differential diagnosis and each histological type has different radiological characteristics [2, 14]. The mesenteric liposarcomas have CT attenuation less than that of muscle and MRI signal intensity similar to that of water. Before contrast enhancement, the myxoid components appeared to be cystic on CT attenuation and MRI signal intensity and they appeared to be solid after contrast enhancement [14]. In our case the CT scan appearance was a well-defined oval hypodense uniform mass with a central rounded higher density soft tissue area within it.

The only curative treatment for a mesenteric liposarcoma consists of a wide excision and clear surgical margins followed by adjuvant radiotherapy in high risk patients [15]. It is reported that neoadjuvant chemotherapy helps in reducing the size of the primary tumour and renders the tumour resectable without the need for en bloc resection of the adjacent organs. However, the role of adjuvant chemotherapy remains unclear [3].

## 4. Take Home Messages

1. Patients with previous history of liposarcoma should be treated with high index of suspicion, even after five years of disease-free followup.
2. CT scan is an ideal investigation to detect any evidence of disease recurrence as well as to identify the possible cause of small bowel obstruction.
3. Followup of these patients in regional sarcoma centres is ideal and research to investigate the role of adjuvant chemotherapy is required.

## 5. Conclusions

Mesenteric liposarcoma is a rare soft tissue malignancy with high risk of metastasis and recurrence. We are adding to the literature the second case of a recurrent mesocolon liposarcoma 6 years after complete excision of the liposarcoma lesion.

## Figures and Tables

**Table 1:** Liposarcoma classification and characteristics according to the World Health Organisation classification of tumours [5].

Type	Incidence	Recurrence	Prognostic factor	Mortality rate	Survival
Atypical lipomatous tumor “ALT”/well differentiated “WD”	40–45%	Lesions located in a surgically amenable soft tissue do not recur following WLE with clear margin	Anatomic locations “deep soft tissue liposarcoma carries high risk”	0% for ALT of extremities to 80% for WD in the retroperitoneum	6–11 years when followed up for 10–20 years
Dedifferentiated	10%	40% local recurrence and 15–20% for distant metastasis	Anatomic locations (retroperitoneum carries the worst clinical behaviour)	28–30% at 5-year followup (this figure is higher at 10–20-year followup)	—
Myxoid	10%	Prone to recur locally and one-third develop metastasis	High histological grade (≥5% RC areas), presence of necrosis, and TP53 overexpression carries unfavourable prognosis	—	—
Pleomorphic	5%	30–50% metastasis rate	Tumour depth, size, >20 mitosis in 10 HPFs, and presence of necrosis carries a worse prognosis	40–50% mortality	Patient dies within a short period of time
Mixed type	Extremely rare	—	—	—	—

**Table 2:** Demographic and clinical characteristics of published cases of mesenteric liposarcomas.

Author	Age/sex	Presentation	Location	Primary/ secondary	Size (cm)	Weight (kg)	Type	Followup	Recurrence
Ishiguro et al. [3]	30 y/M	Abdominal distension	Terminal ileum mesentery and right sided mesocolon	Primary	30 cm	—	Myxoid	26 m	Yes (abdominal)
Nakamura et al. [16]	77 y/F	Fever	Ileocecal mesentery	Primary	10.5 × 7 × 7 cm	—	Pleomorphic	7 m	No
Cha [17]	76 y/F	Abdominal mass and frequent micturition	Small bowel mesentery	Primary	5 × 4.3 × 4.2 cm	—	Well differentiated	—	—
Jukic et al. [4]	77 y/M	Weight loss, oedema, and shortness of breath	Small bowel mesentery	Primary (multiple)	35 × 15 × 15 cm	23.5 kg	Well diff./dediff. and pleomorphic	8 days	RIP
Zhianpour and Sirous [9]	35 y/M	Constipation, weight loss, vomiting, and abdominal distension	Sigmoid mesocolon	Primary	50 × 40 × 10 cm	—	Well differentiated	24 m	No
Benedict [12]	56 y/F	Constipation, belching, and feeling bloated	Transverse mesocolon	Recurrent	12.5 cm (5 in)	—	Low-grade liposarcoma	11 m	No
Núñez Fernández et al. [18]	67 y/F	Abdominal mass	Jejunal mesentery	Primary	8.5 × 7.5 cm	—	Myxoid	12 m	No
Tomita et al. [6]	47 y/F	Abdominal distension, frequent urination, and constipation	Ileal mesentery	Metastatic	28 × 23 × 22 cm	1.8 kg	Myxoid	7 m	Yes (liver and heart)
Pawel et al. [13]	15 y/F	Vomiting and abdominal pain	Small bowel mesentery	Primary	Large “unresectable”	—	Pleomorphic	—	Unresectable tumour
Nagawa et al. [7]	33 y/M	Vomiting and abdominal pain	Ileal mesentery and omentum	Metastatic	8 × 5 × 5 cm “mesenteric” and 13 × 11 × 5 cm “omental”	—	Round cell	1.3 m	RIP Lung, liver, and bone mets
Cerullo et al. [19]	55 y/M	Abdominal distension and weight loss	Mesentery	Primary	40 cm	9 kg	Well differentiated	12 m	No
Yuri et al. [1]	73 y/M	Abdominal mass	Duodenal mesentery	Primary	12.4 × 9.6 cm	0.5 kg	Well differentiated	6 m	No
Hirakoba et al. [14]	65 y/F	Abdominal mass	Jejunal mesentery	Primary	16 × 13 × 9 cm	0.7 kg	Well differentiated	—	—
Jain et al. [15]	50 y/M	Abdominal mass, fever, and weight loss	Jejunal mesentery	Primary	20 × 20 cm	1.8 kg	Pleomorphic	—	—
Goel et al. [10]	48 y/M	Abdominal pain and nausea	Sigmoid mesocolon and mesorectum	Primary	—	—	Well differentiated	—	—
Panagiotopoulos et al. [20]	71 y/M	Abdominal pain and distension	Small bowel mesentery	Recurrent	10 × 9 × 7 cm	—	Dedifferentiated	—	Incomplete resection
Amato et al. [11]	75 y/F	Constipation	Sigmoid mesocolon	Primary	2 cm	—	Well differentiated	24 m	No
Calò et al. [21]	43 y/M	Abdominal pain, change in bowel habit, constipation, dyspeptic syndrome, and meteorism	Small bowel mesentery	Primary	20 × 16 cm	2.1 kg	Well differentiated	33 m	No
Manson [22]	60 y/F	Vomiting, abdominal pain, weight loss, and distension	Small bowel mesentery “ileum”	Primary	—	—	Well differentiated	1 m	No
Current case	41 y/F	Abdominal pain, distension, and vomiting	Mesocolon	Recurrent	12 × 11 × 6 cm	0.3 kg	Myxoid	2 m	Yes (current episode)
